# Readers Respond

**Published:** 2008

**Authors:** N.N. Wig

**Affiliations:** *Professor Emeritus, Psychiatry, PGIMER, Chandigarh, India. E-mail: wignn@yahoo.co.in*

This is in response to the MSM theme monograph *Academia-Industry Symposium 2007: Medical Practice and the Pharmaceutical Industry: And Ever the Duo shall Meet.*

I recall that it was just five years ago when you first wrote to me, informing me about the start of *Mens Sana Monographs*. I was excited because it was something new, something different that was happening in the field of mental health and philosophy in India.

You have come a long way since then. In fact, it is amazing how rapidly MSM has progressed, as testified to by the present theme monograph. Your readers and contributors are no longer confined to India; you are now international in reach and outlook. Please allow me to wholeheartedly congratulate you. It is a remarkable achievement by any standard.

The present volume is excellent in its design and contents. The two editorials by Jerome Kassirer and Joel Lexchin are outstanding and deeply thought provoking. The article by Martin Van Der Weyden is equally rewarding and has very useful information. The main monograph has been superbly, very competently, and comprehensively written by both of you.

In the series “What Medicine Means To Me,” I especially liked the piece by Helen Herrman. Her message of public health psychiatry is particularly relevant for developing countries.

The book review of David Healy’s book by Leemon McHenry is very refreshing and challenging. I congratulate you on your decision to publish it when other journals were still hesitating.

And lastly, the obituary reference to my dear friend Dr. Ravi Kapur by Ajit Bhide and you were so touching; I cried all over again.

So keep up your good work, dear friends. May God bless you and may you both achieve many more successes in your careers.



